# Improved risk-stratification for posterior fossa ependymoma of childhood considering clinical, histological and genetic features – a retrospective analysis of the HIT ependymoma trial cohort

**DOI:** 10.1186/s40478-019-0820-5

**Published:** 2019-11-14

**Authors:** Stephanie T. Jünger, Martin Mynarek, Inken Wohlers, Evelyn Dörner, Anja zur Mühlen, Natalia Velez-Char, Katja von Hoff, Stefan Rutkowski, Monika Warmuth-Metz, Rolf-Dieter Kortmann, Beate Timmermann, Sven Rahmann, Ludger Klein-Hitpass, Andre O. von Bueren, Torsten Pietsch

**Affiliations:** 1 Department of Neuropathology, Institute of Neuropathology, DGNN Brain Tumor Reference Center, University of Bonn, Sigmund-Freud-St. 25, D-53127 Bonn, Germany; 2 Present address: Department of Neurosurgery, University of Cologne Medical Center, Cologne, Germany; 3 Department of Pediatric Hematology and Oncology, University Hospital Hamburg-Eppendorf, Hamburg, Germany; 4 Genome Informatics, Institute of Human Genetics, University Hospital Essen, University of Duisburg-Essen, Essen, Germany; 5 Present address: Group for Medical Systems Biology, Lübeck Institute of Experimental Dermatology and Institute for Cardiogenetics, University of Lübeck, Lübeck, Germany; 6 Institute of Diagnostic and Interventional Neuroradiology, University Hospital Würzburg, Würzburg, Germany; 7 Department of Radiation Oncology, University Hospital Leipzig, Leipzig, Germany; 8 Westdeutsches Protonentherapiezentrum, Essen, Germany; 9 Department of Cell Biology (Tumor Research), University of Duisburg-Essen Medical Center, University Hospital Essen, University of Duisburg-Essen, Essen, Germany; 10 Present address: Division of Pediatric Hematology and Oncology, Department of Pediatrics, Obstetrics and Gynecology, University Hospital of Geneva, Geneva, Switzerland

**Keywords:** Ependymoma, Risk stratification, Posterior fossa, Genetics, Neuropathology

## Abstract

**Introduction:**

Risk stratification of children with ependymomas of the posterior fossa in current therapeutic protocols is mainly based on clinical criteria. We aimed to identify independent outcome predictors for this disease entity by a systematic integrated analysis of clinical, histological and genetic information in a defined cohort of patients treated according to the German HIT protocols.

**Methods:**

Tumor samples of 134 patients aged 0.2–15.9 years treated between 1999 and 2010 according to HIT protocols were analyzed for histological features including mitotic activity, necrosis and vascular proliferation and genomic alterations by SNP and molecular inversion probe analysis. Survival analysis was performed by Kaplan-Meier method with log rank test and multivariate Cox regression analysis.

**Results:**

Residual tumor after surgery, chromosome 1q gain and structural genomic alterations were identified as predictors of significantly shorter event-free (EFS) and overall survival (OS). Furthermore, specific histological features including vascular proliferation, necrosis and high mitotic activity were predictive for shorter OS. Multivariate Cox regression revealed residual tumor, chromosome 1q gain and mitotic activity as independent predictors of both EFS and OS. Using these independent predictors of outcome, we were able to build a 3-tiered risk stratification model that separates patients with standard, intermediate and high risk, and which outperforms current stratification procedures.

**Conclusion:**

The integration of defined clinical, histological and genetic parameters led to an improved risk-stratification model for posterior fossa ependymoma of childhood. After validation in independent cohorts this model may provide the basis for risk-adapted treatment of children with ependymomas of the posterior fossa.

## Introduction

In childhood, ependymoma represents the second most frequent intracranial malignant neoplasm. The most prevalent site is the posterior fossa. Knowledge about the biology of ependymomas has increased substantially through genome-wide expression, methylation and DNA sequence analysis [24]. In particular, although it is believed that all ependymomas are derived from similar subventricular glial progenitor cells, namely radial glial progenitors, their biology and underlying pathogenetic events are different according to their location [7, 19]. While supratentorial ependymomas are characterized by recurrent oncogenic fusions, infratentorial ependymomas can be classified by their epigenetic signatures into two main groups, pediatric-type (PFA) and adult-type (PFB) ependymomas [2, 17, 24]. In childhood, most tumors are PFA showing DNA hypermethylation compared to PFB tumors and loss of the histone3-K27me^3^ mark [12, 16]. These tumors can be further substratified into methylation subgroups, whose clinical relevance is unclear however [15]. Risk stratification in most current clinical trials is mainly based on clinical criteria such as age and extent of resection; some studies have included WHO grading (classic WHO grade II versus anaplastic WHO grade III) in their stratification schemes [13, 14]. Although genetic markers with strong prognostic significance such as gain of the long arm of chromosome 1 (chr. 1q) have been identified, these markers have not been used for improved risk stratification. In this study we systematically analyzed the histology and genetics of infratentorial ependymomas in a defined trial cohort to identify independent risk parameters which add to current patient stratification schemes with the aim to inform future clinical trials.

## Materials and methods

### Patients and tumor material

One hundred and thirty four children with ependymomas WHO grades II and III of the posterior fossa diagnosed between 1999 and 2010 were included in this study if the diagnosis was confirmed by central neuropathological review at the Brain Tumor Reference Center of the DGNN at the Institute of Neuropathology, University of Bonn Medical Center, Germany, clinical follow-up data and formalin-fixed, paraffin embedded (FFPE) material for molecular analysis were available. In addition, fresh-frozen material was available from 31 patients (29 with clinical follow-up data). All examinations were carried out on the basis and according to the legal requirements of the revised Declaration of Helsinki of the World Medical Association in 1983. Informed consent was given at study inclusion by the parents or adolescent patients themselves. Corresponding demographic and clinical data were provided by the HIT study center at the University of Hamburg Medical Center, Hamburg-Eppendorf, Germany. The patients received risk-adapted treatment according to guidelines of the HIT- ependymoma protocols with a combination of radio- and chemotherapy adjusted to age at diagnosis, WHO grading and extent of resection. Between 2001 and 2010, the patients were enrolled into the HIT2000 ependymoma trial (ClinicalTrials.gov NCT00303810). Six patients of our cohort were diagnosed between 7/1999 and 10/2000 before the official start of the study but treated according the same strategy. Patients between 18 months and 4 years received conventional irradiation; older patients were intended to receive hyperfractionated irradiation. HIT-SKK chemotherapy (without intraventricular methotrexate) was administered to all patients before and/or after irradiation except for completely resected WHO grade II tumors. In case of residual tumor, patients were evaluated for potential second-look surgery after each therapy element.

### Neuropathological evaluation

Central neuropathological evaluation was performed at the time of diagnosis at the Brain Tumor Reference Center of the DGNN at the Institute of Neuropathology, University of Bonn Medical Center, Germany. The tumors were classified according to the WHO classification of tumors of the CNS by at least two experienced neuropathologists after evaluation of H&E and immunohistochemically stained slides (GFAP, EMA, Ki-67) [11]. For this study, all cases were re-evaluated to assess mitotic activity, presence of necrosis and vascular endothelial proliferation, as well as any specific histological features. Histopathological evaluation was performed on H&E, Ki-67 (MIB-1, Dako, Glostrup, Denmark) and phospho-histoneH3 (Biocare, Concord, USA) stained sections. The latter antibody marks cells which are in the mitotic phase of the cell cycle. Areas with high Ki-67 index were chosen to determine mitotic activity by counting mitotic figures in ten high power fields (HPF, area of 0.238mm^2^). Trimethylated histone3-Lys27 (H3-K27me^3^) was detected using the rabbit MAb C36B11 (Cell Signaling Technologies, Frankfurt, Germany). Positive endothelial cell nuclei served as internal control for this stain.

### DNA extraction and genome-wide copy number analysis

HE stained sections from FFPE material were examined and only areas with more than 80% tumor content were used for DNA extraction with the QIAamp DNA Mini Kit (Qiagen Hilden, Germany) according to the manufacturer’s instructions. Concentration and purity of the extracted DNA were measured using the Nanodrop 1000 Spectrophotometer at an extinction of 260/280 nm (Thermo Fisher Scientific, Waltham, Massachusetts, USA). The concentration of double-stranded DNA was determined prior to MIP analysis by Picogreen or Qubit methodology (Thermo Fisher). Fresh frozen samples were examined by frozen section to confirm tumor content. DNA was then extracted by phenol-chloroform extraction after proteinase K digestion followed by ethanol precipitation [3]. Subsequently, the DNA was analyzed using Genome-Wide Human SNP, Array 6.0 (Affymetrix, Santa Clara, USA).

To identify copy number gains and losses, we used av. molecular inversion probe (MIP) array including 335,000 inversion probes (Version 2.0, Affymetrix, Santa Clara, USA) with a median probe spacing of 2.4 kb. The MIP array contains probes for 541 frequent somatic cancer mutations and was performed as previously described [22]. All probes contain two genomic homology regions each flanking a SNP site. After annealing to the DNA, the gaps are filled and ligated, followed by exonuclease digestion of remaining non-circularized probes. After cleavage the now inverted probes are amplified by PCR using universal primers. Finally, probes are labelled with fluorescent molecules and hybridized to oligonucleotide chip arrays. Raw MIP data was analyzed using the Nexus Copy Number 7.0 Discovery Edition software (BioDiscovery, El Segundo, USA). BioDiscovery’s SNP-FASST2-Segmentation algorithm was used to make copy number and loss of heterozygosity (LOH) calls. Ploidy was determined by analyzing the allele ratio data from the MIP assay. GISTIC (Genomic Identification of Significant Targets in Cancer) analysis was utilized to identify significant focal chromosomal aberrations from random background (p level: 0.05) [3].

In a next step, ependymomas were assigned to the numerical (only whole chromosomal gains or losses), structural (partial chromosomal alterations, e.g. chromosome 1q gain) or balanced (no chromosomal aberration) genomic group [4, 5].

### mRNA sequencing and expression analysis

RNA was extracted from frozen tumor material using the RNAeasy kit from Qiagen. For the construction of DNA libraries, 500 ng RNA were processed using the TruSeq™ RNA and DNA Library Preparation kit (Illumina, San Diego, California, USA) in accordance with the manufacturer’s protocol. After sequencing on the HiSeq2500 platform (Illumina), sequencing reads were mapped to GRCh37 using Top Hat2 with Ensembl gene annotation and quantified using htseq-count (https://htseq.readthedocs.io). Raw read counts were normalized and variance-stabilized using DESeq [1]. Non-negative matrix factorization using the R package NMF was run to determine two, three, four and five clusters. Further clustering of the samples were obtained using different algorithms and different numbers of genes N, always using the N most variable genes. The following clustering algorithms were used: non-negative matrix factorization, Ward clustering, PAM and K-means clustering. The following values of N were used: 50, 100, 500, 1000, 5000, 10,000, 50,000. A consensus clustering was computed over all algorithms and N combinations. Genes that were differentially expressed between two expression groups were analyzed by Gene Set Enrichment Analysis (GSEA) using SeqGSEA [21] and the following gene sets from the molecular signature database MSigDB [18] C5 (Go gene sets) and C6 (oncogenic gene sets).

### Statistics and survival analysis

Event-free survival (EFS) was defined as the time from surgery to first event (progression or relapse), or date of last follow-up. Survival of patient groups was compared by log-rank test and Kaplan-Meier curves were constructed. Using Cox modelling (Wald backward factor elimination strategy), we tested the independence and prognostic power of clinical, histological and genetic markers. A two-way Spearman correlation was performed using SPSS Statistics 23 (IBM, Bonn, Germany) to identify relationships between individual parameters. In addition, the “Comparison” function of the Nexus Copy Number 7.0 software was used to detect significant differences of genomic alterations between two defined cohorts of tumor samples based on two-tailed Fisher’s Exact test. A *p*-value < 0.05 was considered significant.

## Results

### Patients’ characteristics and neuropathological evaluation

The main demographical, neuropathological and genomic results are summarized in Table 1. The median age at diagnosis was 3.6 years (range 0.2–15.9 years), 42.5% of patients were younger than 3. 61.2% were male and gross-total resection was achieved in approximately 2/3 of patients (64.9%). Only 3 patients (2.2%) had metastatic disease at the time of diagnosis. After a median follow-up of 6.1 years, the whole cohort showed a 5y-EFS of 59.7% and a 5y-OS of 79.1%. Neuropathological classification led to the diagnosis of anaplastic ependymoma WHO grade III in 80.6% of cases. Most tumors showed vascular endothelial proliferation (76.1%) and/ or necrosis (77.6%). Mitotic activity was estimated by counting mitotic figures after phospho-histoneH3 staining. Half of cases had a high mitotic activity (> 10 mitoses per 10 high power fields (HPF)). All cases displayed the characteristic perivascular pseudorosettes, while only 10 cases (7.5%) showed advanced differentiation in form of true ependymal rosettes with central lumen.

**Table 1:** Clinical, histological and genetic characteristics of 134 patients

Parameter		Number of patients	%
		(median; range in years)	
Gender	male	82	*61.2*
	female	52	*38.8*
Age at diagnosis (years)	< 3	57	*42.5*
		(1.72; 0.21–2.97)	
	> 3	77	*57.5*
		(5.09; 3.01–15.90)	
Extent of resection	GTR	87	*64.9*
	STR	47	*35.1*
Initially metastatic disease	yes	3	*2.2*
	no	131	*97.8*
5-year EFS	no event	80	*59.7*
5-year OS	alive	106	*79.1*
WHO-grade	II	26	*19.4*
	III	108	*80.6*
Mitotic activity (mitoses / 10 HPF)	≤ 10	67	
		(4; 0–10)	*50*
	> 10	67	
		(25; 11–169)	*50*
Presence of necrosis	yes	104	*77.6*
	no	30	*22.4*
Presence of vascular proliferation	yes	102	*76.1*
	no	32	*23.9*
Presence of ependymal rosettes	yes	10	*7.5*
	no	124	*92.5*
Genomic group	numerical	37	*27.6*
	balanced	58	*43.3*
	structural	39	*29.1*
Chromosome 1q gain	yes	28	*20.9*
	no	106	*79.1*
Polyploid cytogenetic profile /PFB	yes	6	*4.5*
	no	128	*95.5*

### Genomic alterations in pediatric infratentorial ependymomas

**Fig. 1:**
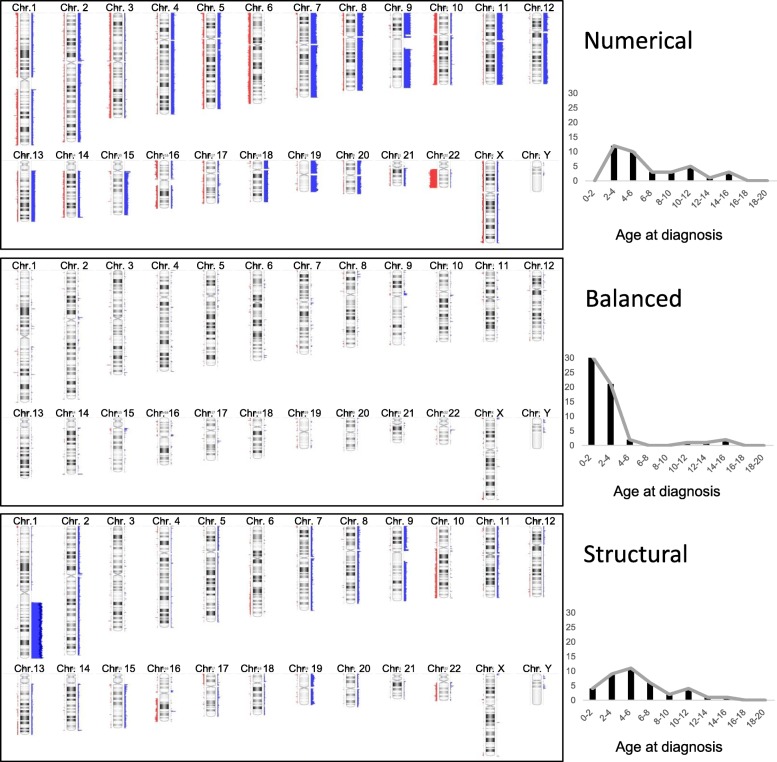
Virtual karyotypes of posterior fossa ependymomas obtained from MIP analysis. Cumulative chromosomal gains are depicted in blue to the right of the chromosome; chromosomal losses are presented in red to the left. Three different genomic groups could be identified, the numerical (top), balanced (middle) and structural (lower panel) genomic group. Patients showed differences in age at diagnosis (right panels)

The most predominant genomic group was the balanced one (43.3%), followed by structural (29.1%) and numerical (27.6%). Belonging to the latter group, 6 cases (4.5%) showing a polyploid cytogenetic profile were identified as adult-type posterior fossa (PFB) tumors (Additional file 1: Figure S1). They showed retained histone3-K27me^3^ and were confirmed as “PFB” tumors by methylation profiling and subsequent epigenetic classification (data not shown). This rare variant occurred only in children older than 6 years, while a balanced genome was predominant in children under 4 years at diagnosis. The most frequent structural aberration in all cases was the occurrence of gain of chr. 1q (20.9%). In the subset of the structural genomic group, 28/39 cases (71.8%) showed chromosome 1q gain. Summary plots and age distribution are illustrated in Fig. 1.

### mRNA expression analysis

RNA sequencing data could be derived from 31 tumors. Multiple cluster analyses including NMF (non-negative matrix factorization) analysis robustly identified two different RNA expression groups (expression group 1, expression group 2) in 29 PFA tumours, see Additional file 2: Figure S2. GSEA analysis identified the gene ontology terms “cell projection part” and “secretin like receptor activity” as enriched among the expression groups (data not shown). When ependymomas with and without chromosome 1q gain were compared, the signature “site of polarized growth” and the oncogenic signature “Hinata NFkB matrix” were found to be enriched.

### Association of clinical, histological and genomic data

Association between pairs of categorical variables were assessed and an association matrix of the clinical, histological and genomic parameters was constructed (Additional file 3: Figure S3). Young age was associated with a balanced genomic profile, the presence of vascular proliferation and WHO grade III. High mitotic activity (both as continuous as well as dichotomous variable) was significantly associated with necrosis and vascular proliferation. Additional significant correlations between the individual parameters are illustrated in Additional file 3: Figure S3.

### Survival analysis – Kaplan Meier analysis and multivariate cox regression

**Fig. 2:**
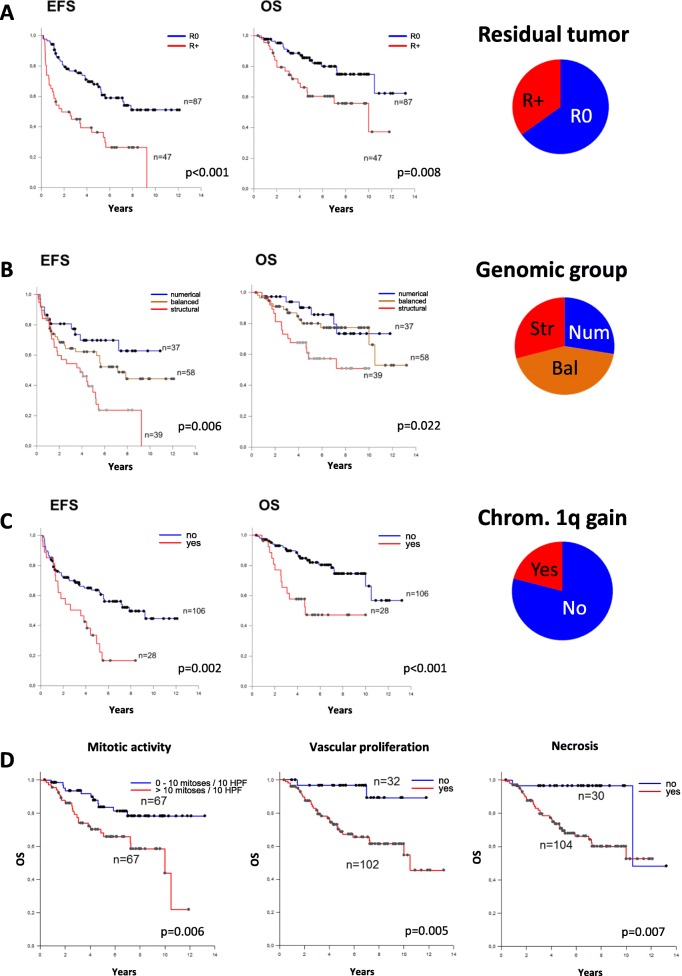
Survival analysis. Kaplan-Meier survival curves show the prognostic impact of **a**, residual tumour; **b**, genomic group, **c**, chromosome 1q gain and **d**, histology (only OS). Coloured pie charts indicate the frequency of the defined subgroups

The presence of residual tumor (35.1%) after surgery was a major adverse prognostic factor both for EFS and OS (Fig. 2a). WHO grading was not found to impact the survival, while individual histological features, in particular mitotic activity (> 10 mitoses /10 HPF), presence of necrosis and/ or vascular endothelial proliferation were associated with worse OS (Fig. 2d). Regarding the genomic groups, patients with tumors in the structural group had worse outcomes (EFS and OS; Fig. 2b). The surrogate marker for the structural group, namely chromosome 1q gain, was highly predictive both for EFS and OS as well (Fig. 2c). In fact, when a genome-wide comparison of genetic alterations was performed between tumors of surviving versus deceased patients, chromosome 1q gain was the only alteration displaying significant difference (Additional file 4: Figure S4). In the subcohort of posterior fossa ependymomas of pediatric type (“PFA”) with fresh frozen material and thus available RNA expression data, expression group 2 showed worse OS (*p* = 0.043) while EFS did not show differences (Additional file 2: Figure S2D). These results were not incorporated into further analysis because only a small fraction of the whole cohort could be studied and comparability would not be granted.

**Fig. 3:**
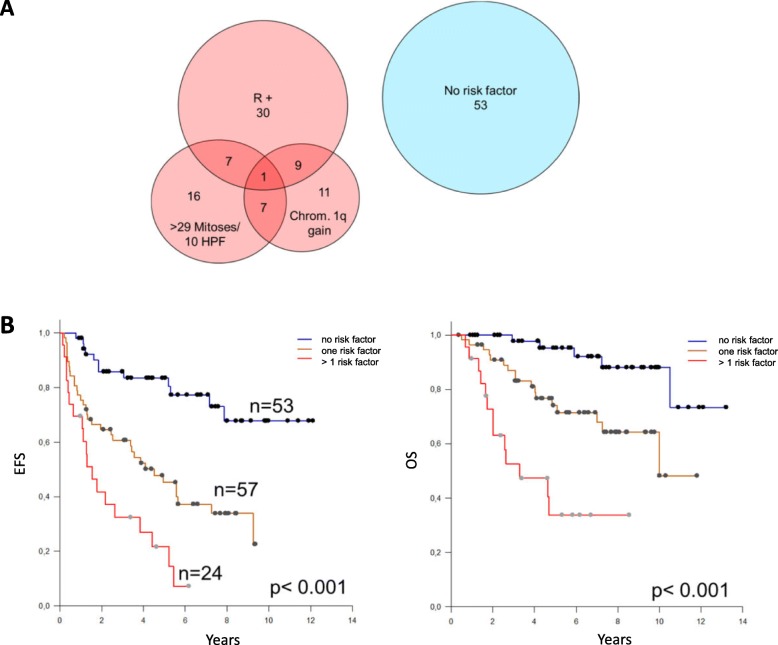
Risk stratification model including the independent parameters residual disease, chromosome 1q gain and mitotic activity. **a** Venn diagram indicating the frequency of these parameters; **b** Kaplan-Meier survival analysis shows different outcomes of the patients with standard, intermediate and high risk. Survival was significantly different between all risk groups for EFS and OS (*p* < 0.001 for all comparisons)

Multivariate Cox regression analysis revealed residual tumor, chromosome 1q gain, and high mitotic activity as independent predictors of EFS and OS (Table 2). When the PFB tumors were removed from the cohort, these three factors again were identified as independent predictors in the remaining 128 cases (data not shown). Using the three most robust independent predictors, we were able to construct a three-tiered risk stratification model (Fig. 3). The three risk groups show statistically different survival when compared with each other (EFS: standard vs intermediate risk, *p* < 0.001; standard vs high risk, p < 0.001; intermediate vs high risk, *p* = 0.007; OS: standard vs intermediate risk, *p* = 0.004; standard vs high risk, *p* < 0.001; intermediate vs high risk, *p* = 0.002). This model integrating clinical (residual tumor after surgery), pathological (mitotic activity) and genetic (chromosome 1q gain) parameters was based on the number of risk factors (none, one risk factor, more than one risk factor). The distribution of cases within the risk groups and calculated survival outcomes are illustrated in Fig. 3. The 5-year EFS/ OS were 84.9% / 96.2%, for the standard, 49.1% / 77.2% for the intermediate and 29.2%/ 45.9% for the high risk group, respectively. This model outperformed a previously published model [9], in which only residual tumor and chromosome 1q gain was considered (Additional file 5: Figure S5). When this model was applied to our data, the following results were found: EFS: standard vs intermediate risk, *p* < 0.001; standard vs high risk, p < 0.001; intermediate vs high risk, *p* = 0.420; OS: standard vs intermediate risk, *p* = 0.004; standard vs high risk, p < 0.001; intermediate vs high risk, *p* = 0.221. Therefore, this model could not identify significant survival differences between the intermediate and high risk group.

**Table 2:** Multivariate Cox-regression analysis. Mitotic activity was analyzed as continuous parameter

**Event-free Survival**				
**Covariate**	**Coefficient**	**StdErr**	**Wald Chi-Square**	**P Value**
**1q Gain**	0.684	0.272	6,295	**0.012**
**Resection**	1.053	0.255	17,072	**<0.001**
**Mitotic activity (cont.)**	0.008	0.003	5.826	**0.016**
**Covariate**	**Hazard Ratio**	**95%Conf-L**	**95%Conf-U**	
1q Gain	1.964	1.161	3.389	
Resection	2.866	1.739	4.723	
Mitotic activity (cont.)	1.008	1.002	1.015	
**Overall Survival**				
**Covariate**	**Coefficient**	**StdErr**	**Wald Chi-Square**	**P Value**
**1q Gain**	0.897	0.374	5.754	**0.016**
**Resection**	1.124	0.381	8.703	**0.003**
**Mitotic activity (cont.)**	0.015	0.005	10.574	**0.001**
Necrosis	0.739	0.763	0.937	0.333
Vascular proliferation	1.431	0.752	3.619	0.057
**Covariate**	**Hazard Ratio**	**95%Conf-L**	**95%Conf-U**	
1q Gain	2.453	1.178	5.107	
Resection	3.078	1.458	6.496	
Mitotic activity (cont.)	1.015	1.006	1.024	
Necrosis	2.094	0.469	9.351	
Vascular proliferation	4.181	0.958	18.253	

## Discussion

The patients analyzed in this study represent a cohort that received standard treatment within the prospective, non-randomized, multicenter HIT2000 ependymoma trial. Demographic and clinical parameters were similar to the recently published AIEOP ependymoma study [13]. Residual disease (36.4% in AIEOP versus 35.1% in this cohort), 5-year EFS (60.9% vs 59.7%) and 5-year OS (77.7% vs 79.1%) were comparable. Although the detailed treatment schedules of irradiation and chemotherapy differed slightly between the two protocols, the overall strategy with stratification by age, extent of resection and WHO grade was very similar. Therefore the analyzed cohort for which clinical follow-up data and material for genomic analysis was available appears to be representative for the spectrum of pediatric posterior fossa ependymoma.

While extensive genetic and biological studies have identified several ependymoma entities, differing in regards to their location / cell of origin, genetic driver mechanisms, biological and clinical behavior, recent clinical treatment strategies still rely on uniform approaches considering clinical factors such as age at diagnosis and extent of resection. Approaches for risk-adapted stratification of children with ependymoma taking into account genetic or biological information are rare, although many studies have been published on possible prognostic biomarkers. However, these have been mainly derived from small single institutional cohorts or from retrospective collections of patients not enrolled in controlled clinical trials. In addition, most studies report on mixed supratentorial / infratentorial cohorts. Few exceptions have identified / confirmed the important prognostic role of residual disease after surgery and the presence of chromosome 1q gain as independent adverse prognostic marker in pediatric ependymoma patients treated in clinical trials [9]. In their study, Kilday *et al*. proposed a risk stratification model integrating these two factors allowing the identification of a low, intermediate and high risk group with respect to EFS depending on absence, presence of one, or presence of both factors. Applying this model to the HIT ependymoma trial cohort, we were able to verify the better EFS of patients lacking both adverse factors, but no significant difference between the “intermediate” and “high risk” group (Additional file 5: Figure S5).

In several larger cohorts, due to restrictions in quality or quantity of FFPE material genetic analysis has been restricted to FISH analysis of predefined regions such as chromosome 1q. Most genome-wide genetic or epigenetic studies have been applied to retrospective collections of material, derived from inhomogeneously treated patients. In this study, mostly small quantities of archival FFPE material were available. Therefore, we applied a sensitive method for the quantitative evaluation of genome-wide copy number alterations and allelic distribution by molecular inversion probe analysis [23]. Using this reliable method, we were able to retrieve genome-wide, high-resolution, quantitative data on copy number alterations and allelic distribution. We could assign each tumor to one of the three genomic groups - “numerical”, “balanced” and “structural” - and found characteristic differences concerning age distribution with balanced genomes most frequently found in infant patients (Fig. 1). In addition, together with demonstration of retained H3-K27me^3^ we identified six “PFB” ependymomas in this cohort of 134 patients. These few patients showed polyploid genomes and occurred at an older age; all patients were at least 6 years at time of diagnosis (Additional file 1: Figure S1). Therefore, this epigenetic subgroup typically occurring in adults does not seem to play a significant role in pediatric ependymoma. In a recently published series of pediatric ependymomas, PFB ependymoma did not show a different outcome compared to PFA ependymoma [14]. The epigenetically defined “PFA” tumors representing the vast majority of ependymomas of the posterior fossa in children, have recently been shown to further substratify in epigenetic sub-subgroups [15]. The clinical relevance remains unclear, however, because there was no outcome difference between these variants, with the exception of a minor group of Otx2 positive cases with good outcome. To search for clinically meaningful variants of pediatric ependymoma of the posterior fossa, we performed RNA sequencing and unsupervised clustering in 29 cases for which fresh frozen material was available. Using multiple methods to perform clustering, we identified two robust expression groups (Additional file 2: Figure S2). GSEA analysis identified different signaling and oncogenic pathways. *OTX2* positive cases, however, were not found in this cohort, and PFB-type tumors were excluded from the latter analysis. Analyzing the biological differences of the two robust expression groups of “PFA” tumors is subject of ongoing studies. Interestingly, patients with tumors of the expression group 2 showed worse OS. Because of the limited number of cases available, the interpretation of this data is limited.

We were able to retrieve genome-wide information on genomic alterations in all tumors of our cohort, even in cases with minute FFPE material. In line with the findings of Dyer *et al.* [5] and Korshunov *et al*. [10] the genomic group with a “structural” profile showed significantly worse outcome compared to the other groups. The most frequent alteration in this latter group of ependymomas was gain of chr. 1q, occurring in 72% of cases of structural genomic group. Consequently, chr. 1q overlaps with the structural genomic group identified by Dyer* et al*. as adverse prognostic marker. In fact, in univariate analysis, both “1q gain” and “structural genomic group” were associated to worse outcome in our cohort (Fig. 2). Because of the interdependency of the two parameters and the fact that chromosome 1q gain was found as the only chromosomal copy number alteration with significant difference in frequency between alive and deceased patients (Additional file 4: Figure S4), we included chromosome 1q gain in further multivariate analyses and development of risk-stratification models.

WHO grading (grade II versus grade III) has been found to be predictive in various studies [10, 14, 20] but lacked significance in some defined pediatric cohorts [6]. This might be explicable by inhomogenous composition of study cohorts and different treatment approaches or by unprecise grading criteria available for ependymomas leading to increased inter-observer variability between different neuropathologists [6, 7]. In particular, the importance of necrosis, vascular proliferation and the extent of foci with high cellularity for grading have been under debate. Therefore, we analyzed the individual histopathological parameters separately in order to be able to calculate their individual value as prognostic markers. By using this approach, it turned out that in univariate analysis presence of necrosis, vascular proliferation and high mitotic activity indicated unfavorable OS in this trial cohort. In contrast to the strong outcome predictors “extent of resection” and “chromosome 1q gain”, these parameters also showed only a trend for worse EFS. Interestingly, the histological parameters high mitotic activity, necrosis and vascular proliferation were frequently found associated (Suppl. Figure 3.) in line with findings reported previously [6–8]. Multivariate COX regression analysis identified mitotic activity, completeness of resection and chromosome 1q gain as independent prognostic factors for EFS and OS (Table 2). Mitotic activity was identified as independent risk factor when tested as categorical as well as continuous parameter. In a next step we addressed the questions if the addition of the histological parameter “high mitotic activity” could improve the risk-stratification model previously published by Kilday *et al*. [9] Since mitotic activity represented a continuous parameter we aimed to identify the optimal cut-off for this parameter in the model that was identified as > 29 mitotic figures / 10 HPF. Incorporating the parameters “residual tumor”, “chromosome 1q gain” and “high mitotic activity” we were able to build a three-tiered risk model with a standard (no risk factor, 39.6% of patients), intermediate (one of the three risk factor present; 42.5% of patients) and high risk group (more than one risk factor present, 17.9% of patients) with significantly different EFS and OS (Fig. 3). The standard -risk group of patients with a 5-y EFS of 84.9% indicate that not all “PFA” ependymomas represent a lethal disease as postulated previously [12]. This finding is in line with recent data by Merchant *et al*. who found a 5-y EFS of 81.5% in PFA tumors without 1q gain [14]. However, long-term follow-up data will be required to discuss a possible de-escalation of the treatment of these patients. In contrast, patients with tumors of the high risk group may require more intensive/ different treatment approaches or may be selected for experimental treatment strategies.

This novel risk-stratification for the first time includes clinical, genetic and histological features and suggests the inclusion of different levels of information for optimal allocation of patients to risk-strata and treatment algorithms. The three-tiered risk stratification model has to be confirmed in independent trial cohorts. After validation, it may provide a basis for future risk-adapted treatment of children with infratentorial ependymoma.

## Supplementary information

**Additional file 1: Figure S1.** Virtual karyotypes of posterior fossa ependymomas obtained from MIP analysis. Cumulative chromosomal gains are depicted in blue to the right of the chromosome; cumulative chromosomal losses are presented in red to the left. A, summary plot of PFA tumours; B, of PFB tumours showing characteristic polyploidy. Patients showed differences in age at diagnosis (right panels).

**Additional file 2: Figure S2.** Unsupervised clustering based on RNA sequencing of 29 posterior fossa “PFA” ependymomas was performed by various algorithms using different numbers of most variable genes. A, NMF analysis with different numbers of groups. B, NMF rank survey identified a best model with two groups. C, two robust RNA expression subgroups were identified by various alternative clustering methods using different numbers of most variable genes; “consensus” specifies the most frequent cluster assignment for every tumour. D, worse overall, but no worse event-free survival of patients with expression group 2 tumors.

**Additional file 3: Figure S3.** Association of clinical, histological and genomic data. A two-way Spearman correlation was performed to identify relationships between individual characteristics. Green boxes indicate significant positive association between two parameters, red boxes indicate significant negative association.

**Additional file 4: Figure S4.** Frequency of chromosome 1q gain in tumours of alive versus deceased patients with infratentorial ependymomas. A two-sided Fisher’s Exact test was used for detection of significant differences in the genome, e.g. to identify altered genomic regions between two defined cohorts of patients. Gain of chromosomal regions of chromosome 1q was the only significant difference.

**Additional file 5: Figure S5.** Risk stratification model including the independent parameters residual disease and chromosome 1q gain in this cohort. Survival analysis by log-rank test shows different outcomes of the patients with standard versus the other groups, but not between intermediate and high risk.

## Data Availability

The datasets during and/or analysed during the current study available from the corresponding author upon reasonable request.
