# Supplementary material for: Improved risk-stratification for posterior fossa ependymoma of childhood considering clinical, histological and genetic features – a retrospective analysis of the HIT ependymoma trial cohort
Source: Acta Neuropathol Commun. 2019 Nov 14;7:181. doi: 10.1186/s40478-019-0820-5 (PMC6857225; doi:10.1186/s40478-019-0820-5)
Supplement: Supplementary file 1 — Additional file 1: Figure S1. Virtual karyotypes of posterior fossa ependymomas obtained from MIP analysis. Cumulative chromosomal gains are depicted in blue to the right of the chromosome; cumulative chromosomal losses are presented in red to the left. A, summary plot of PFA tumours; B, of PFB tumours showing characteristic polyploidy. Patients showed differences in age at diagnosis (right panels). [file 40478_2019_820_MOESM1_ESM.pptx]

## Slide 1
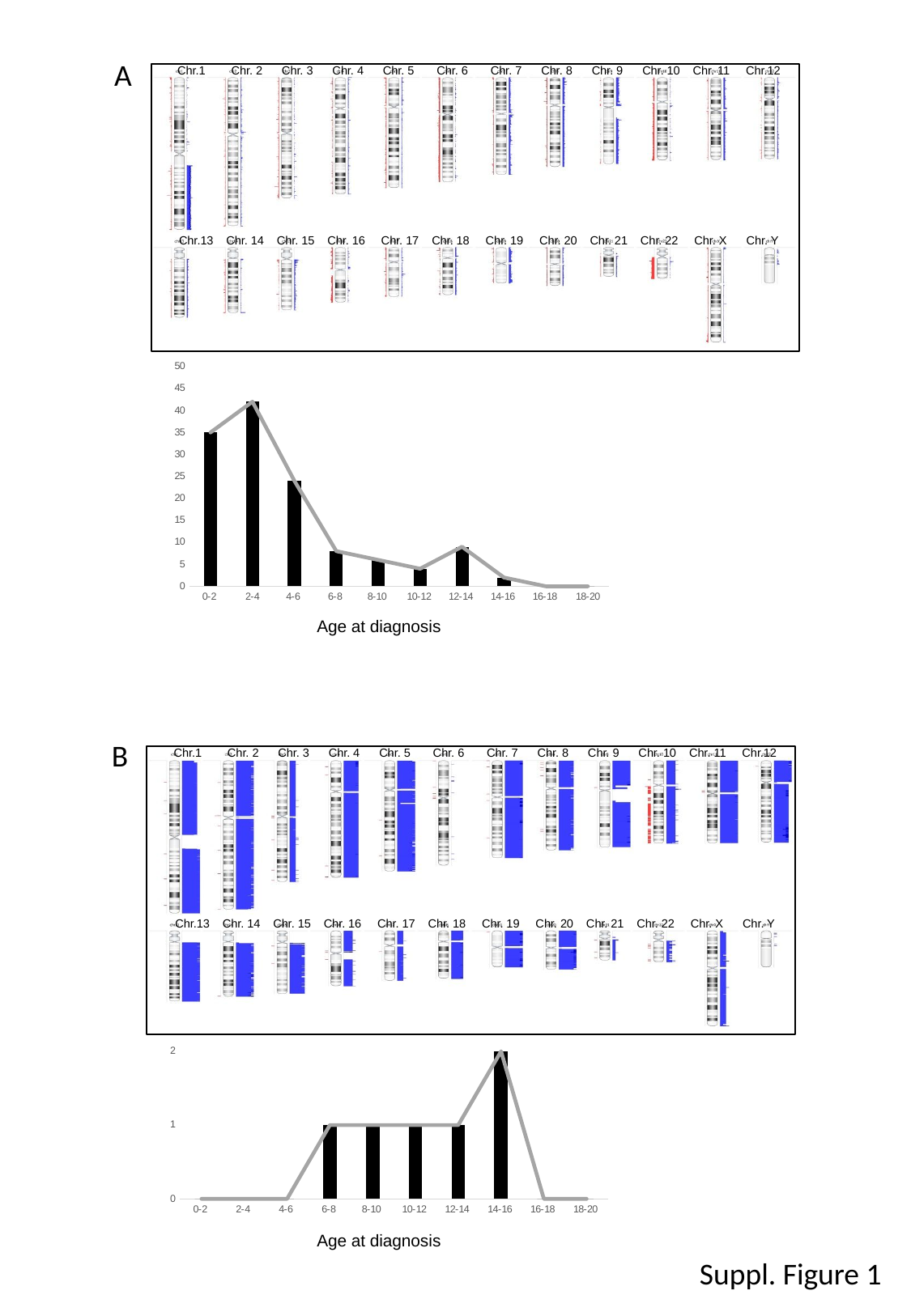

A
Chr.1 Chr. 2 Chr. 3 Chr. 4 Chr. 5 Chr. 6 Chr. 7 Chr. 8 Chr. 9 Chr. 10 Chr. 11 Chr.12
Chr.13 Chr. 14 Chr. 15 Chr. 16 Chr. 17 Chr. 18 Chr. 19 Chr. 20 Chr. 21 Chr. 22 Chr. X Chr. Y
[unsupported chart]
Age at diagnosis
B
Chr.1 Chr. 2 Chr. 3 Chr. 4 Chr. 5 Chr. 6 Chr. 7 Chr. 8 Chr. 9 Chr. 10 Chr. 11 Chr.12
Chr.13 Chr. 14 Chr. 15 Chr. 16 Chr. 17 Chr. 18 Chr. 19 Chr. 20 Chr. 21 Chr. 22 Chr. X Chr. Y
[unsupported chart]
Age at diagnosis
Suppl. Figure 1
