# Supplementary figures and images for: Improved risk-stratification for posterior fossa ependymoma of childhood considering clinical, histological and genetic features – a retrospective analysis of the HIT ependymoma trial cohort
Source: Acta Neuropathol Commun. 2019 Nov 14;7:181. doi: 10.1186/s40478-019-0820-5 (PMC6857225; doi:10.1186/s40478-019-0820-5)

## Slide 1
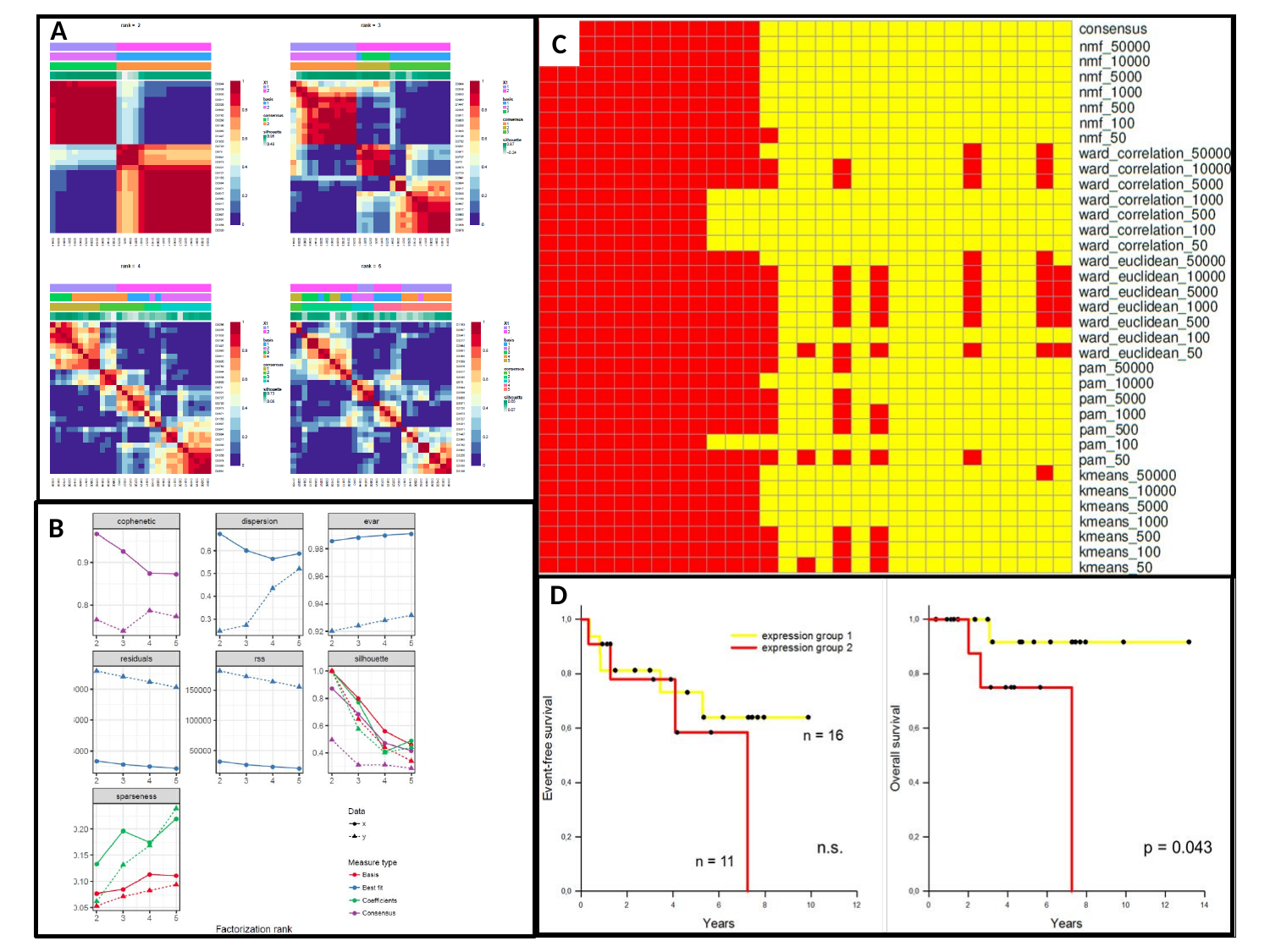

A
C
B
D

Supplement: Supplementary file 2 — Additional file 2: Figure S2. Unsupervised clustering based on RNA sequencing of 29 posterior fossa “PFA” ependymomas was performed by various algorithms using different numbers of most variable genes. A, NMF analysis with different numbers of groups. B, NMF rank survey identified a best model with two groups. C, two robust RNA expression subgroups were identified by various alternative clustering methods using different numbers of most variable genes; “consensus” specifies the most frequent cluster assignment for every tumour. D, worse overall, but no worse event-free survival of patients with expression group 2 tumors. [file 40478_2019_820_MOESM2_ESM.pptx]

## Slide 1
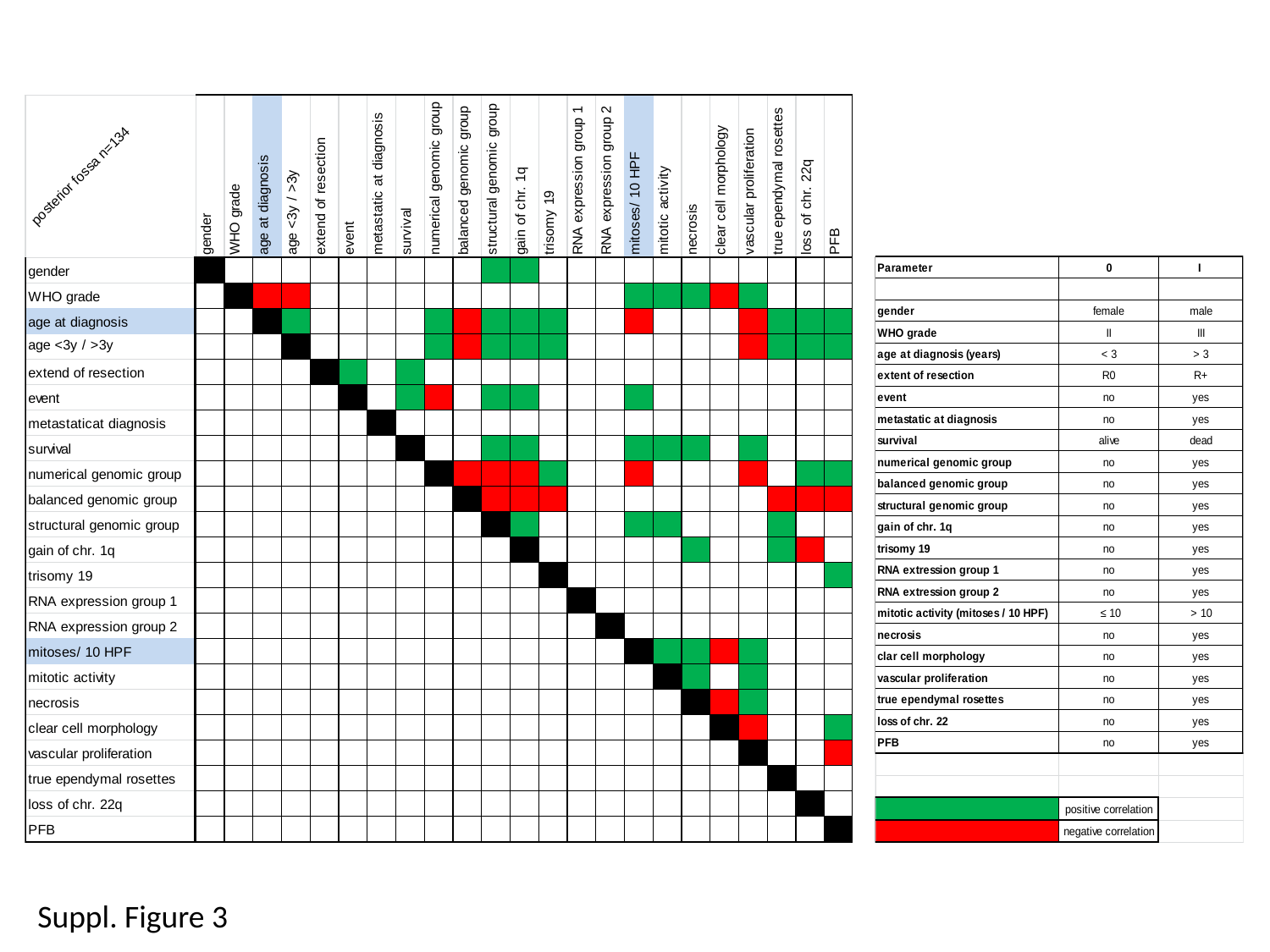

Suppl. Figure 3

Supplement: Supplementary file 3 — Additional file 3: Figure S3. Association of clinical, histological and genomic data. A two-way Spearman correlation was performed to identify relationships between individual characteristics. Green boxes indicate significant positive association between two parameters, red boxes indicate significant negative association. [file 40478_2019_820_MOESM3_ESM.pptx]

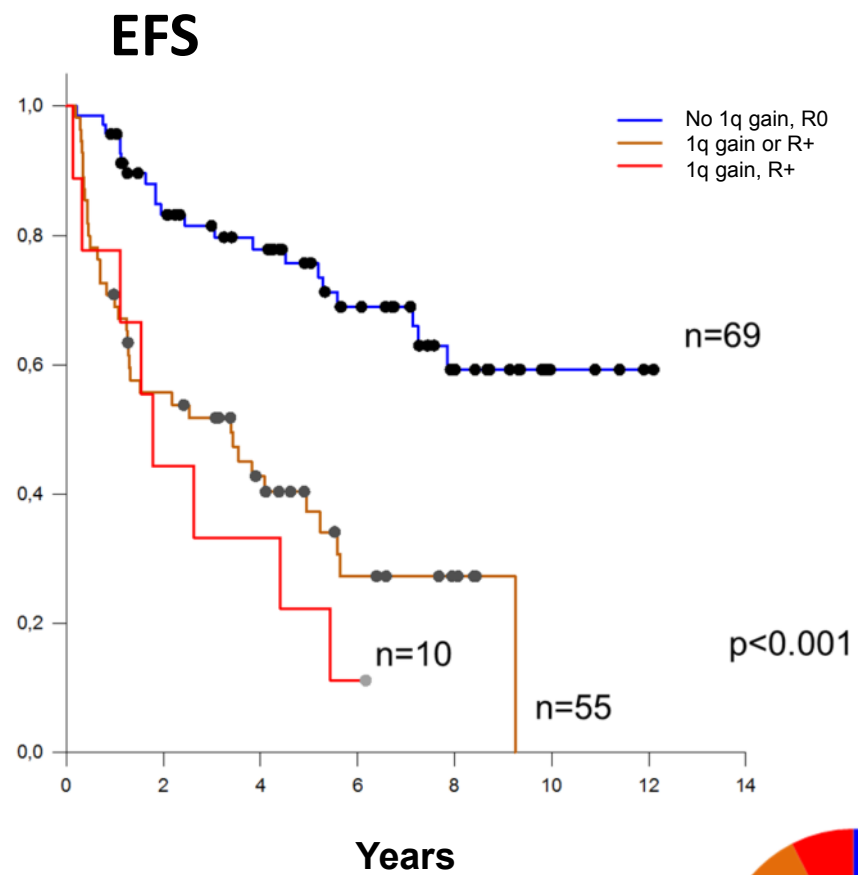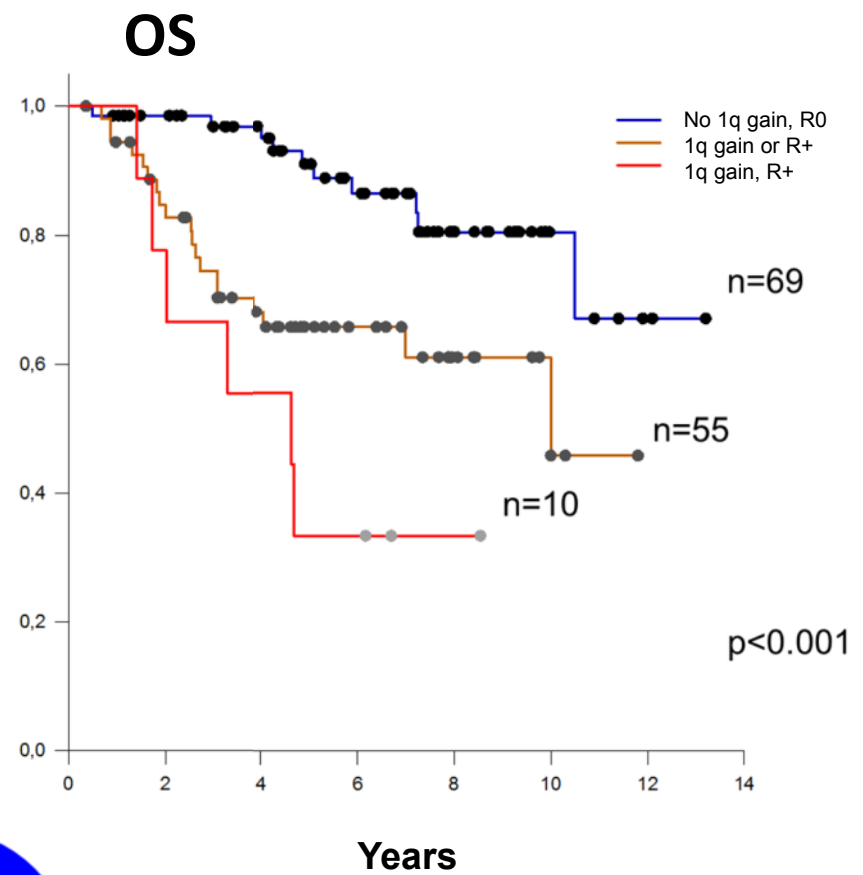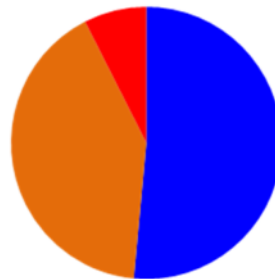

Suppl. Figure 5

Supplement: Supplementary file 5 — Additional file 5: Figure S5. Risk stratification model including the independent parameters residual disease and chromosome 1q gain in this cohort. Survival analysis by log-rank test shows different outcomes of the patients with standard versus the other groups, but not between intermediate and high risk. [file 40478_2019_820_MOESM5_ESM.pdf]
